# Amendment of saline-alkali soils promotes the formation and stability of iron-bound organic carbon

**DOI:** 10.1016/j.isci.2025.114314

**Published:** 2025-12-02

**Authors:** Shuhan Wang, Xueqin Ren, Tairan Zhou, Yun Zhang, Shuwen Hu, Biao Zhu

**Affiliations:** 1 College of Resources and Environmental Sciences, China Agricultural University, Beijing 100193, China; 2 State Key Laboratory for Vegetation Structure, Function and Construction (VegLab), Ministry of Education Key Laboratory for Earth Surface Processes, and College of Urban and Environmental Sciences, Peking University, Beijing, China

**Keywords:** earth sciences, soil science, applied sciences

## Abstract

Iron oxides protect soil organic carbon (SOC) over the long term, but the effects of saline-alkali soil amendments on iron-bound organic carbon (Fe-OC) remain unclear. Using data from Northeast China, this study examined the effects of desulfurized gypsum and polyaspartic acid calcium (PASP-Ca) on Fe-OC. Compared to the control, Fe-OC increased by 2.7 times with desulfurized gypsum and 3.9 times with PASP-Ca. Both amendments shifted the association of Fe-OC from adsorption to co-precipitation, thereby enhancing the formation and stability of Fe-OC and consequently promoting SOC sequestration. Both measures also facilitated the transformation of free iron minerals into complexed forms, reducing microbial carbon binding while maintaining the pattern of preferential binding of iron minerals with aromatic-C. Key factors driving these changes include improved soil nutrients, water content, and microorganisms, along with reduced soil pH. This study offers valuable insights into the effects of saline-alkali soil amendments on Fe-OC and SOC stabilization.

## Highlights

• Soil improvement increases soil nutrients and water content but decreases pH
• Soil improvement enhances the formation and stability of Fe-OC
• Improvements reduce the preferential binding of Fe with microbial-derived C
• Changes in soil traits and microbes drive variations in Fe-OC and Fe oxides

## Graphical abstract

**Figure:**
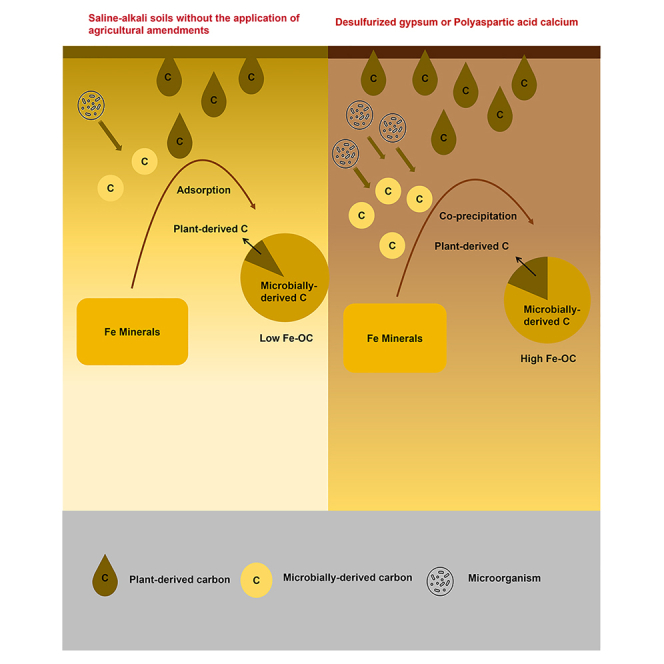

## Introduction

The characteristics of saline-alkali soil include high pH and sodium concentration.^1^ According to FAO statistics, the global area of saline-alkali soil totals approximately 1.381 billion hectares, accounting for roughly 10.7% of the world’s agricultural land. Furthermore, in the context of global climate change, soil salinization is becoming increasingly severe.^2^ Consequently, saline-alkali soil amendments are crucial for ensuring global food security and increasing soil carbon storage. Soil organic carbon (SOC) serves as a critical indicator for evaluating the effectiveness of amendment measures. The existing measures typically contribute to an increase in SOC storage.^3^ SOC plays an essential role in global carbon cycling and climate regulation. Therefore, evaluating the impact of saline-alkali soil amendments on SOC holds greater significance.

This categorization of SOC into particulate organic carbon (POC) and mineral-associated organic carbon (MAOC) facilitates a more precise assessment of the contribution of SOC to the global carbon cycle.^4^^,^^5^^,^^6^ POC consists of partially decomposed plant material and its decomposition byproducts, exhibiting a relatively short mean residence time.^4^^,^^6^^,^^7^ The estimated global mean turnover time of POC is 23 years^6^ In contrast, MAOC is protected by chemical bonds with minerals and represents a stable carbon form, exhibiting longer turnover times and higher chemical stability.^6^^,^^8^ The estimated global mean turnover time of MAOC is 129 years^6^ Fe-bound organic carbon (Fe-OC) is a vital constituent of MAOC and is formed by adsorption and/or co-precipitation.^7^^,^^9^ Considering the critical role of Fe-OC in global carbon sequestration and the mitigation of soil CO_2_ emissions, numerous scholars have conducted extensive research on Fe-OC across various ecosystems globally, including forests, grasslands, farmlands, and permafrost regions.^10^ However, despite saline-alkali soils constituting a substantial proportion of terrestrial areas, studies focusing on Fe-OC in saline-alkali soils remain scarce.

The formation and stability of Fe-OC are influenced by a multitude of abiotic and biotic factors.^10^^,^^11^^,^^12^^,^^13^ Saline-alkali soil amendments will result in significant alterations in soil physicochemical properties, microbial communities, and enzyme activities.^4^^,^^14^ These changes are expected to influence the forms and activity of iron minerals, alter the absorption capacity of organic carbon by Fe oxides, and modify the selectivity of Fe oxides toward various functional groups, ultimately impacting the formation and stability of Fe-OC. However, the impact of saline-alkali soil amendments on Fe-OC remains unexplored. The absence of such research hinders our comprehensive understanding of soil carbon cycling. Therefore, it is of considerable importance to investigate the effects of saline-alkali soil amendments on Fe-OC.

Conventional chemical amendments, such as desulfurized gypsum, have been extensively utilized in the reclamation of saline-alkali soils. However, they have a high cost, a large dosage, and easily cause secondary pollution of soil and water.^15^^,^^16^ Environment-friendly amendments, such as polyaspartic acid calcium (PASP-Ca), are a non-toxic and natural biodegradable polymeric amino acid material. It is gradually replacing conventional desulfurized gypsum to alleviate soil salinization and enhance plant yields.^17^^,^^18^

Desulfurized gypsum and PASP-Ca are currently commonly used in saline-alkali soil amendments; thus, this study aims to evaluate the impact of the application of these two amendments on Fe-OC in saline-alkali soils. The specific objectives are as follows: (1) conduct a systematic investigation into the impact of saline-alkali soil amendment on the formation and stability of Fe-OC; (2) assess the relationship between Fe-OC and biotic and abiotic factors, elucidating the underlying mechanisms; (3) explore the effects of saline-alkali soil amendments on the transformation of iron minerals and the associated potential mechanisms.

## Results

### The impact of amendment measures on soil nutrients, soil physicochemical properties, and microorganisms

**Figure 1:**
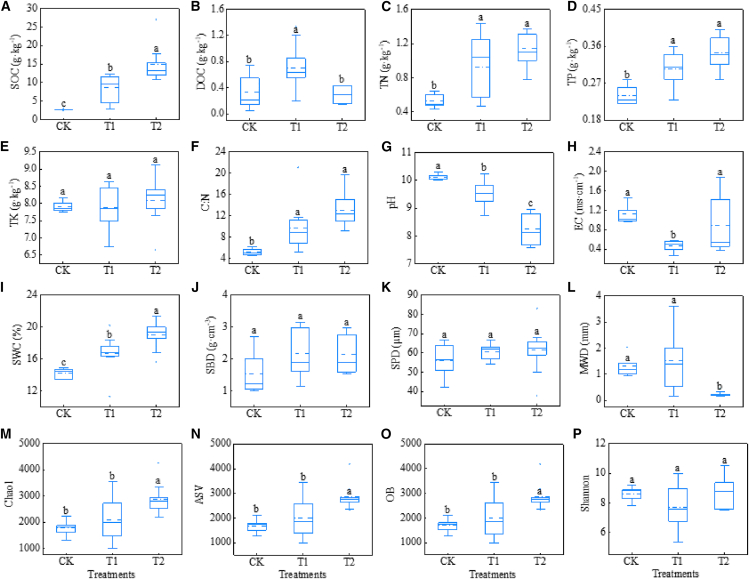
Variations in soil nutrients, physicochemical properties, and microbial richness and diversity across different treatments (A–P) (A) Soil organic carbon (SOC); (B) dissolved organic carbon (DOC); (C) total nitrogen (TN); (D) total phosphorus (TP); (E) total potassium (TK); (F) C: N; (G) pH; (H) EC; (I) soil water content (SWC); (J) soil bulk density (SBD); (K) soil particle diameter (SPD); (L) mean weight diameter (MWD); (M) Chao1; (N) amplicon sequence variants (ASVs); (O) observed species (OB); and (P) Shannon index. Each boxplot shows the average (solid line), minimum, and maximum values. CK is the control treatment, T1 is the improvement measure with the application of desulfurized gypsum to the saline soils, and T2 is the improvement measure with the application of PASP-Ca to the saline soils. The upper and lower boundaries of the box in the figure represent the third quartile (Q3) and the first quartile (Q1), respectively. The solid line within the box denotes the median, while the dashed line indicates the mean. The distance between the upper and lower whiskers, represented by the short horizontal lines outside the box, corresponds to 1.5 times the interquartile range (IQR = Q3 - Q1). In the CK treatment, each indicator is based on 5 data points, whereas in the T1 and T2 treatments, each indicator is derived from 10 data points. Different characters represent a significant difference at *p* < 0.05.

The SOC contents in the CK, T1, and T2 treatments were 2.71 g/kg, 8.61 g/kg, and 14.82 k/kg, respectively (Figure 1A). The SOC content increased by 218% under T1 treatment and by 447% under T2 treatment. However, the effects of the two amendment measures on soil DOC were inconsistent. Compared with the CK treatment, the T1 treatment increased the DOC content from 0.34 g/kg to 0.70 g/kg, resulting in a significant enhancement in content. (*p* < 0.05), whereas the T2 treatment did not show any statistically significant changes (*p* > 0.05) (Figure 1B). The TN content increased by 73.58% and 115.47% under the T1 and T2 treatments, respectively (*p* < 0.05, Figure 1C), while the TP content increased by 26.67% and 43.23%, respectively (*p* < 0.05, Figure 1D), but there was no significant effect on TK contents (*p* > 0.05, Figure 1E). The C: N ratios increased from 5.20 to 9.70 (*p* < 0.05) under the T1 treatment and to 13.03 (*p* < 0.05) under the T2 treatment, respectively (Figure 1F).

The two measures markedly decreased soil pH (*p* < 0.05 for T1 and *p* < 0.05 for T2) with a reduction of 0.6 units in the T1 treatment and 1.9 units in the T2 treatment (Figure 1G). The effects of the two measures on EC in soils were inconsistent. Compared to the CK treatment, the T1 treatment reduced EC by 0.65 units. (*p* < 0.05), whereas the T2 treatment exerted an insignificant effect (*p* > 0.05) (Figure 1H). The two measures led to a substantial increase in SWC (*p* < 0.05 for T1 and *p* < 0.05 for T2) with an increasing of 2.51 units in the T1 treatment and 4.84 units in the T2 treatment (Figure 1I), and the increase in SWC in the T2 treatment was significantly higher than that in the T1 treatment (*p* < 0.05). The two measures had an insignificant effect on SBD and SPD (*p* > 0.05) (Figures 1J and 1K). The impact of amendments on MWD in soils was inconsistent across different measures. MWD in the T1 treatment did not show a significant change (*p* > 0.05), but it significantly decreased by 1.08 units in the T2 treatment (*p* < 0.05) (Figure 1L).

Compared to the CK, the Shannon index did not exhibit significant changes in either the T1 or T2 treatments (*p* > 0.05) (Figure 1P). Additionally, the ASV, Chao1, and OB indices in the T1 treatment showed no significant differences compared to the control (*p* > 0.05 for all indices) (Figures 1M–1O). However, these three indices were significantly elevated in the T2 treatment (*p* < 0.05 for all indices) (Figures 1M–1O).

### The impact of amendment measures on iron minerals

**Figure 2:**
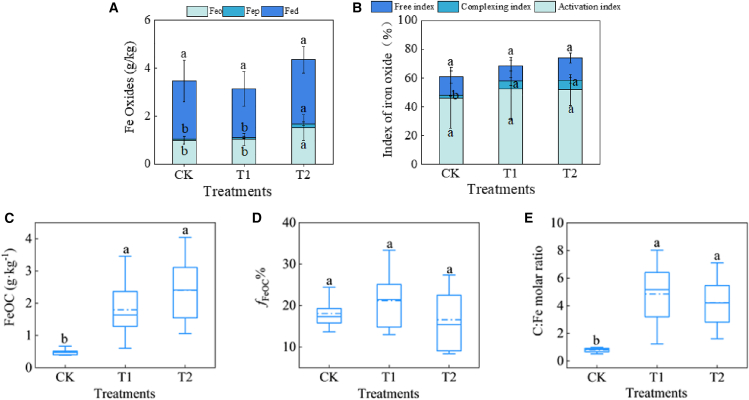
Variations in properties of iron oxides and Fe-OC across different treatments (A) Contents of different forms of iron oxides. (B) Index of iron oxides. (C) Fe-OC content. (D) Proportion of Fe-OC to SOC (*f*_FeOC_%). (E) C: Fe molar ratio of Fe-OC. For Figure 2 (A) and (B), data are represented as mean ± SD. For Figure 2 (C), (D), and (E), the upper and lower boundaries of the box represent Q3 and Q1, respectively. The solid line and dashed lines within the box denote the median and the mean, respectively. The distance between the upper and lower whiskers, represented by the short horizontal lines outside the box, corresponds to 1.5 IQR. In the CK treatment, each indicator is based on 5 data points, whereas in the T1 and T2 treatments, each indicator is derived from 10 data points. Different characters represent a significant difference at *p* < 0.05.

No significant changes were observed in the contents of Feo, Fep, and Fed in the T1 treatment relative to those in the CK treatment (*p* > 0.05 for all) (Figure 2A). Conversely, the T2 treatment increased the Feo content from 0.91 to 1.50 (*p* < 0.05) and the Fep content from 0.045 to 0.18 (*p* < 0.05), while the content of Fed remained unchanged (*p* > 0.05) (Figure 2A). In the T1 and T2 treatments, the complexation index increased from 1.66% to 5.08% and 6.59%, respectively (*p* < 0.05), whereas the Fe-free and Fe-activation index showed no significant change in all treatments (*p* > 0.05) (Figure 2B).

### The impact of amendment measures on the iron-bound organic carbon and the C: Fe molar ratio

The content of Fe-OC and *f*_Fe-OC%_ were 0.49 g/kg and 18.09%, 1.80 g/kg and 21.19%, and 2.40 g/kg and 16.55% in the CK, T1, and T2 treatments, respectively (Figures 2C and 2D). Compared to the CK treatment, Fe-OC content in the T1 and T2 treatments increased by 2.7 and 3.9 times, respectively, but no significant alteration in *f*_Fe-OC%_ (*p* > 0.05). The C: Fe molar ratio of Fe-OC was 0.79, 4.85, and 4.22 in the CK, T1, and T2 treatments, respectively (Figure 2E). Saline-alkali soil improvements significantly increased the C: Fe molar ratio (*p* < 0.05).

### The impact of amendment measures on the δ^13^C of soil organic carbon and iron-bound organic carbon

**Figure 3:**
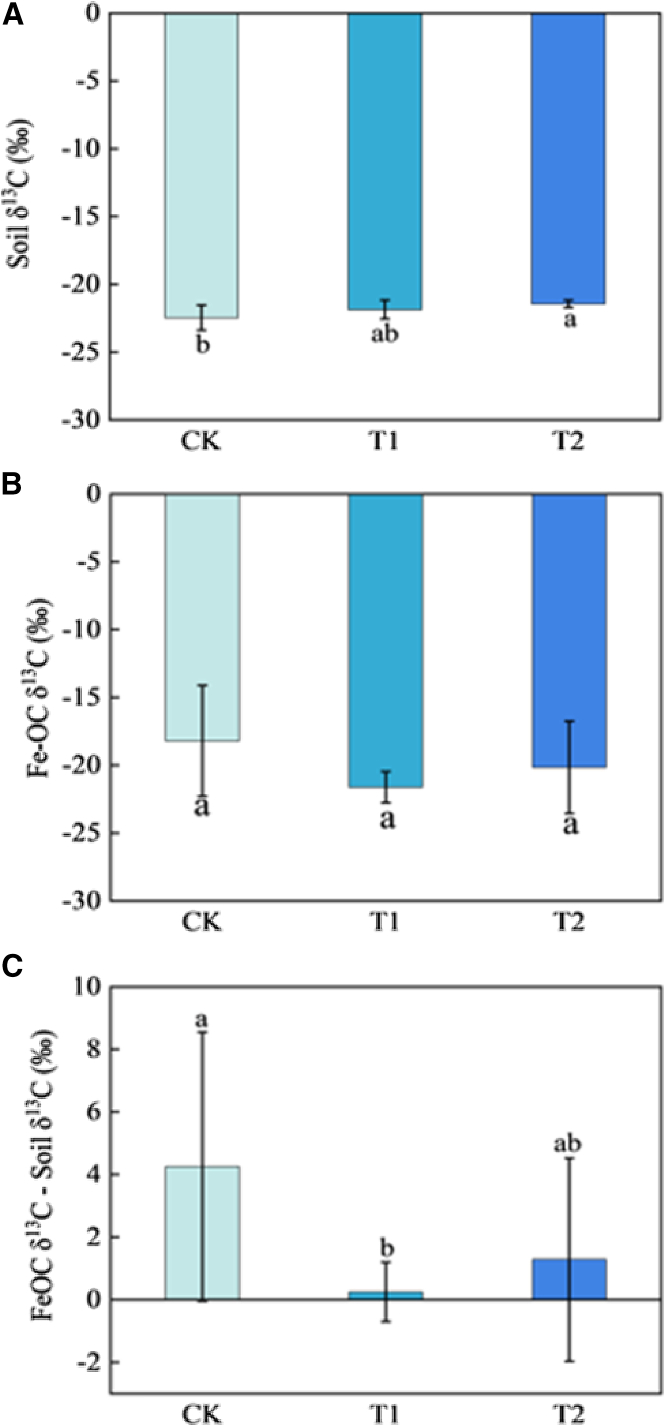
Variations in δ^13^C values across different treatments (A) δ^13^C values of bulk soil (soil δ^13^C). (B) δ^13^C values of Fe-OC (Fe-OC δ^13^C). (C) The δ^13^C difference between Fe-OC and bulk soil. Data are represented as mean ± 1 sd. In the CK treatment, each indicator is based on 5 data points, whereas in the T1 and T2 treatments, each indicator is derived from 10 data points. Different characters represent a significant difference at *p* < 0.05.

The mean δ^13^C value of SOC (SOC δ^13^C) was **-**22.46‰, −21.86‰, and −21.45‰ in the CK, T1 and T2 treatments, respectively. Thus, compared with the CK treatment, the SOC δ^13^C values in T1 and T2 treatments increased by 0.6‰ and 1.01‰, respectively (Figure 3A). The mean δ^13^C value of Fe-OC (Fe-OC δ^13^C) was **−**18.20‰, −21.62‰, and −20.15‰ in the CK, T1, and T2 treatments, respectively, indicating more ^13^C enrichment in the Fe-OC than the corresponding SOC across all treatments. The δ^13^C difference between Fe-OC and SOC was 4.25‰, 0.25‰, and 1.29‰ for the CK, T1, and T2 treatments, respectively (Figures 3B and 3C).

### The impact of amendment measures on the selective adsorption of soil organic carbon by iron oxides

**Figure 4:**
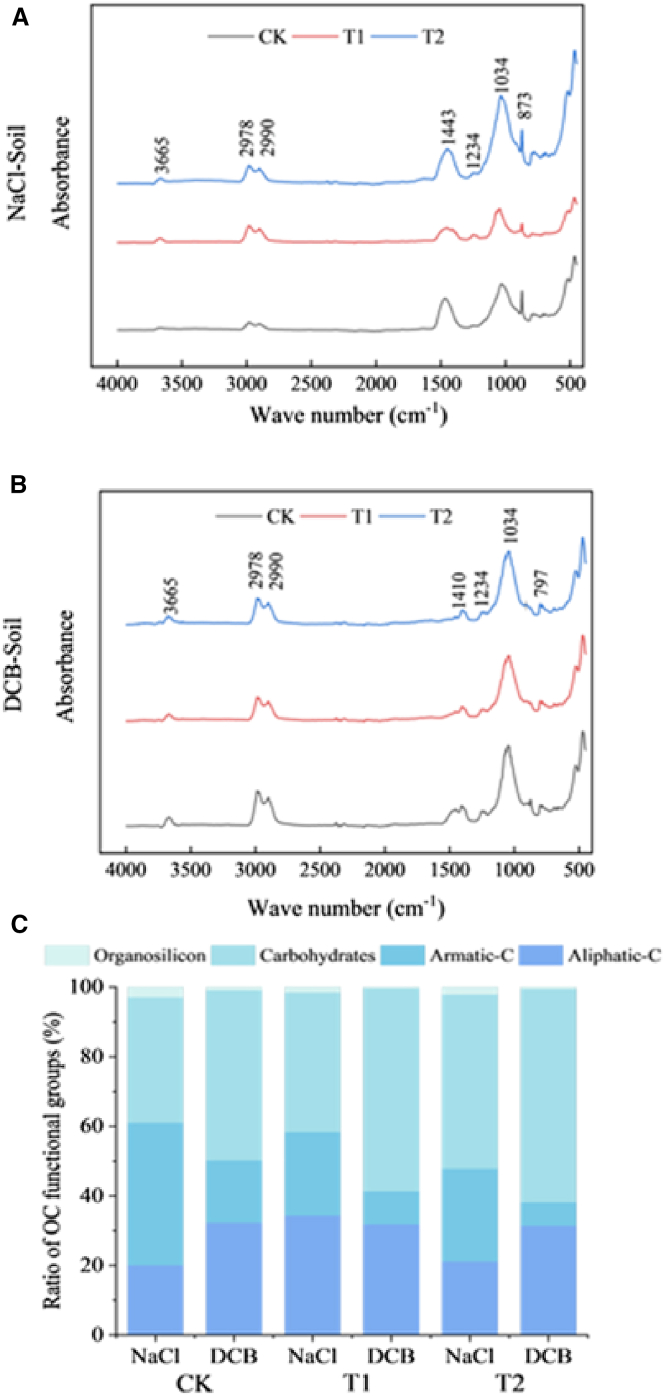
FTIR spectroscopy plot and the relative content of OC functional groups (A) FTIR spectroscopy of soil treated with NaCl. (B) FTIR spectroscopy of soil treated with DCB. (C) The relative content of OC functional groups. In C, data are represented as a mean.

The characteristic peaks observed at 3665 cm^−1^ in the soil samples treated with NaCl and DCB were crystal water expansion in clay minerals. The peaks at 2978 cm^−1^ and 2990 cm^−1^ were attributed to aliphatic-C. The peaks around 1443 cm^−1^ and 1234 cm^−1^ were indicative of aromatic-C. The peak at 1034 cm^−1^ represented carbohydrates. The peaks at 797 cm^−1^ and 873 cm^−1^ were mainly associated with the symmetrical stretching vibrations of O-Si-O bonds in quartz or the stretching of Si-*O*-Al and Si-O bonds in bentonite (Figures 4A and 4B). Compared to the soil treated with NaCl, the soil treated with DCB exhibited a significant decrease in the relative content of aromatic-C across all three treatments, while the relative content of carbohydrates increased significantly. Following DCB treatment, the relative content of aliphatic-C increased in both CK and T2 treatments, whereas it decreased in the T1 treatment (Figure 4C).

### The relationships between different iron indices and abiotic and biotic variables

**Figure 5:**
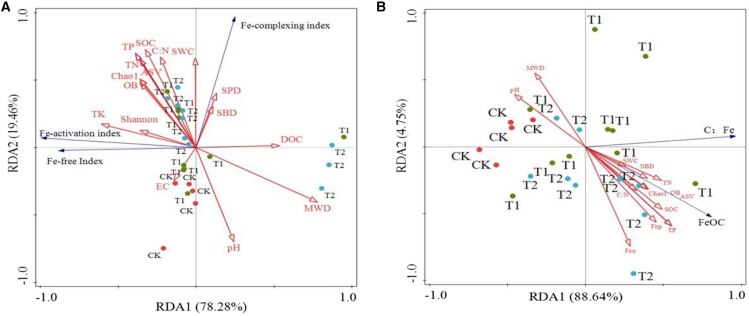
Results of redundancy analysis (A) Three Fe indices in soil. (B) Fe-OC contents and C: Fe molar ratio of Fe-OC. In A, the blue vectors represent the three Fe indices, while the red vectors represent the biotic and abiotic factors. In B, the blue line denotes Fe-OC and C: Fe molar ratio, while the red line indicates soil biotic and abiotic factors.

There is no significant correlation between the Fe-activation index and the measured soil abiotic and biotic variables (Figure S1). The Fe-complexing index was positively correlated with SOC (*p* < 0.05), C: N (*p* < 0.05), TN (*p* < 0.05) and SWC (*p* < 0.05), negatively with soil pH (*p* < 0.05), and not with DOC, EC, TP, TK, SBD, SPD, MWD, ASV, Chao1, Shannon, and OB (*p* > 0.05 for all) (Figure S1). The Fe-free index was positively correlated with SOC (*p* < 0.05), EC (*p* < 0.05), TK (*p* < 0.05), negatively with MWD (*p* < 0.05), and not with DOC, pH, C: N, TN, TP, SWC, SBD, SPD, ASV, Chao1, Shannon, and OB (*p* > 0.05 for all) (Figure S1). The RDA analysis suggested that these abiotic and biotic variables explained 97.74% of the variance in three kinds of Fe indices, with Axes 1 and 2 accounting for 78.28% and 19.46%, respectively (Figure 5A). SWC exhibited the strongest positive correlation with the Fe-complexing index, while TK showed the strongest positive correlation with Fe-activation index and free index. Conversely, pH demonstrated the strongest negative correlation with Fe-complexing index. whereas MWD had the strongest negative correlation with Fe-activation index and free index (Figure 5A).

### The relationships between iron-bound organic carbon contents and the abiotic and biotic variables

**Figure 6:**
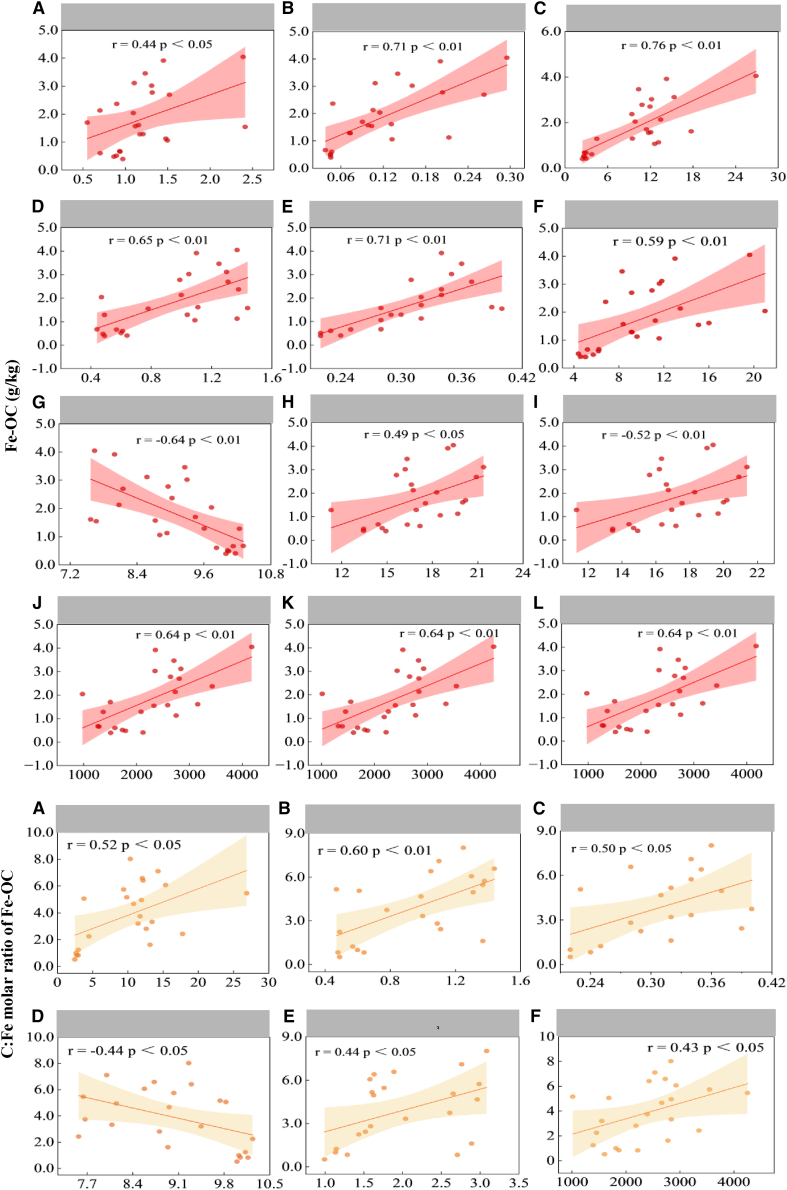
Correlation analysis of Fe-OC contents and C: Fe molar ratio of Fe-OC with soil biotic and abiotic factors The upper figures (A–L) show the correlations between Fe-OC and Feo (A), Fep (B), SOC (C), TN (D), TP (E), C:N (F), pH (G), SWC (H), MWD (J), ASV (K), and Chao1 (L). The lower figures (A–F) show the correlations between C:Fe molar ratio of Fe-OC and SOC (A), TN (B), TP (C), pH (D), SBD (E), and Chao1 (F). Each indicator contains 20 data points (i.e., *n* = 20). A *p* value of <0.05 is considered to indicate a significant correlation. The curve shown in the figure has a 95% confidence level.

The Fe-OC exhibited positive correlations with Feo (*p* < 0.05), Fep (*p* < 0.05), SOC (*p* < 0.05), C: N ratio (*p* < 0.05), TN (*p* < 0.05), TP (*p* < 0.05), SWC (*p* < 0.05), MWD (*p* < 0.05), ASV (*p* < 0.05), Chao1 (*p* < 0.05), OB (*p* < 0.05), a negative correlation with pH (*p* < 0.05) (Figure 6), and no correlation with Fed, EC, SBD, and SPD (*p* > 0.05). The results of RDA analysis indicated that TP exhibited the strongest positive correlation with Fe-OC, while pH demonstrated the strongest negative correlation with Fe-OC (Figure 5B).

### The relationship between the C: Fe molar ratio of iron-bound organic carbon and the abiotic and biotic variables

The molar ratio of C: Fe showed positive correlation with SOC (*p* < 0.05), TN (*p* < 0.05), TP (*p* < 0.05), SBD (*p* < 0.05) and Chao1 (*p* < 0.05), a negative correlation with soil pH (*p* < 0.05) (Figure 6), and no correlation with Feo, Fep, Fed, C: N ratio, TK, EC, SWC, SBD, SPD, and MWD (*p* > 0.05 for all). RDA analysis suggested that TP and pH showed the strongest positive correlation and negative correlation with the C: Fe molar ratio, respectively (Figure 5B).

## Discussion

### The impact of amendment measures on the transformation of iron minerals and potential mechanisms

The forms of iron oxide in the soil include free iron (Fed), amorphous iron (Feo), and complexed iron (Fep). Feo is a poorly crystalline iron oxide and has a large specific surface area.^19^ Fep is also a poorly crystalline iron oxide and usually forms complexes with organic substances, especially humic acids, and its concentration is frequently positively correlated with SOC.

Under certain conditions, the various forms of iron oxide can transform into each other. The complexing index reflects the extent of transformation from free iron to complexed iron in the soil. Our findings demonstrate that the two amendment measures facilitated the transformation of free iron minerals into complexed forms (Figures 2A and 2B), suggesting an enhanced association between iron and humic acids. The study revealed a positive correlation between the complexing index and soil water content (SWC), while indicating a negative correlation with pH. The positive relationship between the complexing index and SWC can potentially be attributed to the fact that an increase in SWC promotes the migration and dissolution of soil humic acids. The influence of pH on the solubility and availability of Fe may account for the strong negative correlation between pH and complexing index. In alkaline soils, iron ions are prone to forming insoluble iron hydroxide and carbonate precipitates, which reduces iron availability.^20^ However, decreased soil pH enhances iron solubility and availability. Consequently, the elevation of SWC and the reduction in pH value, which were brought about by the application of the amendment, jointly facilitated the complexation of iron oxides and humic acids.

### The impact of amendment measures on the iron-bound organic carbon and potential mechanisms

The significant rise in Fe-OC can primarily be attributed to the marked increase in SOC, which provides a greater abundance of OC substrates capable of combining with iron minerals. As a result, a significant positive correlation between Fe-OC and SOC was observed (Figure 6). The positive correlation between Fe-OC and TN, as well as TP, may be attributed to the elevated SOC levels (Figure 6) because the significant increases in TN and TP in the improved soil can enhance plant growth, leading to greater plant-derived organic carbon input into the soil, which consequently boosts SOC levels. The study revealed that there was a significant negative correlation between soil pH and Fe-OC contents (Figure 6). This finding aligns with the observation of previous research.^13^ Two potential mechanisms may account for the strong negative correlation between Fe-OC and pH. First, decreased pH enhances the solubility of Fe^3+^, thereby facilitating the formation of organo-iron associations via adsorption and/or co-precipitation reactions.^21^ Second, iron oxide minerals exhibit variations in surface charge as a function of pH, with typical point of zero charge (PZC) values ranging from pH 7 to 9.^9^^,^^22^ As soil pH decreases below the PZC, the surfaces of iron minerals become increasingly protonated, acquiring a greater net positive charge. This positively charged surface facilitates the strong sorption of negatively charged organic molecules. Ligand exchange between carboxyl/hydroxyl functional groups of organic carbon and hydroxylated iron oxide surfaces is likely a dominant mechanism responsible for Fe-associated carbon formation.^23^^,^^24^ In addition to the aforementioned mechanisms, the application of soil amendments led to a marked decrease in soil pH (Figure 1), which is conducive to plant growth and consequently resulted in greater SOC accumulation. This provided an abundant source of organic carbon for the association with iron minerals. Figure 6 illustrates that Feo and Fep are significantly positively correlated with Fe-OC content. This finding is consistent with the result of a previous study that poorly crystalline iron oxides can adsorb more OC than highly crystalline iron oxides.^25^ This discrepancy can be attributed primarily to the larger specific surface area and higher chelating capacity of poorly crystalline iron oxides compared to their crystalline counterparts.^19^

Microorganisms exert both positive and negative influences on Fe-OC content. On the positive side, increased microbial richness facilitates the formation of Feo and Fep, thereby enhancing OC binding. Conversely, microbial catalysis of Fe(II) oxidation or Fe(III) reduction can lead to the dissociation of Fe-OC complexes.^26^^,^^27^ The observed positive correlation suggests that, in this experiment, the beneficial effects of microorganisms on Fe-OC content outweigh their adverse effects.

Following the application of soil amendments, enhanced SWC was observed (Figure 1), which favors plant growth and subsequently contributes to an increase in SOC. This improved edaphic environment also promotes microbial proliferation. As previously discussed, the concurrent increases in SOC and microbial richness facilitate the formation of Fe-OC. Consequently, a positive correlation between Fe-OC content and SWC was observed (Figure 6). Furthermore, the increase in macro-aggregates enhances the physical protection of organic carbon, thereby contributing to the stabilization of Fe-OC complexes. As a result, a positive correlation between MWD and Fe-OC content was evident (Figure 6).

The *f*_FeOC%_ values obtained in this study are comparable to those reported in a previous research,^28^ indicating that Fe-OC accounted for approximately 22% of farmland organic carbon. However, they are significantly higher than those reported in another previous study,^29^ where the proportion of Fe-OC ranged from approximately 4.0%–7.5%. Notably, the saline-alkali soil amendments did not significantly alter the *f*_FeOC%_. This is attributed to the concurrent significant increases in both Fe-OC and SOC contents following the amendment, thereby maintaining a similar *f*_FeOC%_ across all three treatments.

### The impact of amendment measures on the binding mode of organic carbon and iron oxides

The C: Fe molar ratio of Fe-OC reflects the binding mode of organic carbon and Fe oxides.^29^^,^^30^^,^^31^ When the ratio is less than 1, Fe oxides are combined with OC through sorption. When the ratio ranges from 1 to 6, there are two binding modes of sorption and co-precipitation between Fe oxides and OC. The stability of Fe-OC formed via co-precipitation is significantly higher compared to that formed through sorption.^30^^,^^32^^,^^33^ In this study, the C: Fe molar ratio in the saline soils was less than 1, indicating that Fe oxides were predominantly associated with OC through sorption. Following the saline-alkali soil amendment, the ratios increased to 4.22 and 4.85, respectively, significantly surpassing the maximum sorption capacity of Fe oxides. This suggests that the combination of OC and Fe oxides was primarily characterized by co-precipitation, with adsorption playing a relatively minor role. The result indicates that soil amendments increased the affinity between Fe oxides and OC in the soil, thereby favoring soil carbon sequestration.

The binding affinity between Fe oxides and OC was influenced by multiple abiotic factors (Figures 5 and 6). Specifically, a decrease in pH or an increase in SOC, TN, and TP intensified the binding strength (Figure 6). Moreover, TP and pH are identified as predominant influencing factors (Figure 5B). Currently, there is limited research on how environmental variables affect the binding mode of Fe and OC. The negative correlation observed in this study between pH and the C: Fe molar ratio of Fe-OC supports the argument that a decrease in soil pH enhances the co-precipitation reaction between Fe oxides and SOC.^34^ However, a study conducted in Guangdong Province, China, where the soil pH is 5.21, showed that the C: Fe molar ratio was less than 0.5.^35^ This observation indicates that even in acidic soils, iron minerals are bound to OC by adsorption, which seemed to contradict the above argument. Such inconsistencies or contradictions highlight the uncertainty regarding the effect of soil pH on the binding affinity between Fe oxides and OC. Additionally, the underlying mechanisms driving the positive correlation between the C: Fe molar ratio of Fe-OC and SOC, TN, and TP remain unclear and warrant further investigation.

### Impact of amendment measures on the carbon source of iron-bound organic carbon

Carbon isotope fractionation occurs during the plant-derived C (i.e., new C) is decomposed into SOC, resulting in ^13^C enrichment in the microbial residues and metabolites (i.e., microbial-derived C or older C); thus, the δ^13^C value of the microbial-derived C is more positive than that of the plant-derived C.^36^ The Fe-OC δ^13^C was greater than the corresponding SOC δ^13^C across all treatments (Figures 3A and 3B), indicating that iron minerals preferentially combine with older C to form Fe-OC complexes. Since an increase in the proportion of older C within the Fe-OC complex leads to a higher Fe-OC δ^13^C value, thereby resulting in a more significant δ^13^C difference between Fe-OC and SOC, Figure 3C suggests that while the soil amendment did not alter the overall pattern of preferential binding of iron minerals with older carbon, the extent of this preferential binding was diminished, particularly the treatment with added desulfurized gypsum.

### Impact of amendment measures on the selectivity of iron oxides for organic carbon adsorption

The adsorption of OC by Fe oxides is selective due to OC’s distinct molecular structures and functional groups.^37^^,^^38^ The OC content in the Fe-OC is the difference between the OC content of the soil after DCB treatment and that of the soil after NaCl treatment; consequently, the functional group exhibiting the most significant difference is the one that is predominantly adsorbed by the iron oxides.^10^ Figure 4 suggests that iron minerals exhibited preferential adsorption for aromatic-C, while their adsorption capacity for carbohydrates was relatively weaker. In conclusion, the soil improvement did not alter the selective adsorption behavior of iron minerals toward aromatic carbon and carbohydrates, moreover, compared to the soils treated with NaCl, the relative content of aliphatic-C in soils treated with DCB increased in both the CK and T2 treatments but decreased in the T1 treatment (Figure 4). This indicates that the association capacity of Fe minerals with aliphatic-C in soils is relatively weak. However, the addition of desulfurized gypsum to the soils enhanced this capacity. Despite a few individual studies reporting inconsistent or even contrary findings,^10^ the preferential adsorption of aromatic-C by iron minerals, as well as their relatively weaker affinity for carbohydrates and aliphatic-C, has been substantiated by numerous previous investigations.^32^^,^^38^^,^^39^ For instance, the aromatic-C is more readily adsorbed and co-precipitated by iron oxides compared to aliphatic-C, while aliphatic-C is preferentially released during iron reduction.^32^^,^^38^ The underlying mechanisms for the preferential adsorption of aromatic C by iron minerals remain unclear. This phenomenon can be attributed to electron donor-acceptor interactions between aromatic-C and iron oxides.^40^^,^^41^ Furthermore, A prior study proposed a “layer-by-layer onion” model to elucidate the formation of co-precipitates involving iron minerals and different OC functional groups.^42^ According to this model, aromatic groups, which are preferentially protected, form the initial inner layer of the “onion” through strong specific interactions with iron oxides, providing an absorptive surface where phenolic and aliphatic compounds subsequently bind, resulting in alternating layers within the OC-Fe colloid.

### Limitations of the study

Although based on two amendment measures implemented in Northeast China, these findings point to a potential general mechanism: effective saline-alkali soil amendment consistently enhances soil nutrient status and reduces pH, thereby promoting the formation of iron-bound organic carbon (Fe-OC) and contributing to carbon sequestration. However, given the diversity of currently used soil amendments, the variability of saline-alkali soil types, and the significant differences in climatic and environmental conditions across saline-alkali regions, the conclusion that the amendment of saline-alkali soil promotes Fe-OC formation and carbon sequestration requires further validation. This generalization should be rigorously assessed using data from diverse amendment materials, different types of saline-alkali soils, and multiple geographic regions. Therefore, additional systematic research is warranted.

## Resource availability

### Lead contact

Further information and requests for resources and reagents should be directed to and will be fulfilled by the lead contact, Shuwen Hu (shuwenhu@cau.edu.cn).

### Materials availability

This study did not generate new unique reagents.

### Data and code availability

Raw data were deposited on Mendeley at https://data.mendeley.com/datasets/4v5654vwyz/2. This study did not use any code. Any additional information required to reanalyze the data reported in this article is available from the lead contact upon request.

## Acknowledgments

This work was supported by the 10.13039/501100012166National Key Research and Development Program of China (2023YFD150050305) and the “Double First-Class” project of the 10.13039/501100002338Ministry of Education of China (2024AC008).

## Author contributions

Conceptualization, S.W.H., X.Q.R., and S.H.W.; methodology, S.H.W.; investigation, S.H.W., T.R.Z., and Y.Z. writing – original draft, S.W.H.; writing – review and editing, S.W.H. and B.Z.; funding acquisition, S.W. H.; resources, S.W. H.; supervision, S.W. H., and X.Q. R.

## Declaration of interests

The authors declare no competing interests.

## STAR★Methods

### Key resources table

**Table:** 

REAGENT or RESOURCE	SOURCE	IDENTIFIER
**Chemical reagent and Kit**		

Sodium hydrosulfite	China Agricultural University	N/A
Sodium citrate	China Agricultural University	N/A
Sodium chloride	China Agricultural University	N/A
Sodium bicarbonate	China Agricultural University	N/A
Hydroxylamine hydrochloride	China Agricultural University	N/A
Phenanthroline	China Agricultural University	N/A
Sodium acetate	China Agricultural University	N/A
Sodium pyrophosphate	China Agricultural University	N/A
Ammonium oxalate	China Agricultural University	N/A
Oxalic acid	China Agricultural University	N/A
Soil DNA Kit	Omega Bio-Tek	https://omegabiotek.com/

**Software and algorithms**		

IBM SPSS Statistics 27	SPSS, USA	https://www.ibm.com/cn-zh/products/spss-statistics
OriginLab 2022	OriginLab, USA	https://www.originlab.com/

**Deposited data**		

Raw data	This study	Mendeley dataset https://data.mendeley.com/datasets/4v5654vwyz/2

**Instrument**		

Spectrophotometer	Shimadzu, Japan	https://www.shimadzu.com/
TOC analyzer	Shimadzu, Japan	https://www.shimadzu.com/
PCR	Thermo Fisher Scientific, USA	https://www.thermofisher.cn/cn/zh/home.html
Delta Plus XP mass spectrometer	Thermo Scientific, Germany	https://www.thermofisher.cn/cn/zh/home/promo/campaigns.html
ATR-FTIR	Bruker, USA	https://www.bruker.com/zh.html

### Method details

#### Study area

The experiment site is situated in Northeast China (124°28′E, 45°00′N). This region falls within the soda-saline soil of the Songnen Plain. The experiment site features a temperate monsoon climate with a mean annual precipitation of 450 mm and a mean annual temperature of 4.5°C. The yearly evaporation is about 1,200 mm. The combination of high evaporation rates and inadequate soil drainage results in serious accumulation of salinity. Before improvement, the soil was classified as severely salinized soil. Based on the soil texture classification of the United States Department of Agriculture (USDA), the soil texture was categorized as sandy loam.

#### Experimental treatments

Chemical amendment, desulfurized gypsum (T1), and environmentally-friendly amendment, PASP-Ca (T2), were separately applied to improve the saline soils. The T1 improvement encompasses 1 ha, including one-year (T1-1, 0.5 ha) and three-year improvements (T1-3, 0.5 ha). Similarly, the T2 improvement also spans 1 ha, comprising one-year (T2-1, 0.5 ha) and three-year improvements (T2-3, 0.5 ha). The desulfurized gypsum sourced from Changchun power station, and the PASP-Ca is a product independently developed by our research group.^17^ Based on the previous experiments, the field application dosages of desulfurized gypsum and PASP-Ca were both 7,500 kg/ha. A control check (CK) refers to the treatment without amendment with an area of 0.5 ha.

#### Soil sampling

The sampling procedure was conducted in April 2023. Soil samples were collected from the topsoil layer at a depth of 0–20 cm. Five sampling points were set following the five-point sampling method. Specifically, five samples were collected in the CK treatment, and ten samples were collected respectively in the T1 and T2 treatments. Among them, five samples were collected in each of the one-year and three-year improvement treatments. We used shovels and ring knives to collect samples. The samples collected using shovels were air-dried naturally, and the soils were sieved through a 2-mm mesh for the analysis of soil physicochemical properties. The samples collected using a ring knife were for the determination of soil bulk density (SBD) and soil water-stable aggregates.

### Quantification and statistical analysis

#### Soil physicochemical property analysis

Soil pH and electrical conductivity (EC) were measured at the soil-to-distilled water ratio of 1:5 (w/v) using a pH and EC analysis meter (Leici, DZS-708L, China). Soil organic carbon (SOC) was measured by a TOC analyzer (Shimadzu, TOC-L, Japan), total soil total nitrogen (TN) was measured by Kjeldahl Azotometer (FOSS, Kjeltec8400, Denmark), soil total phosphorus (TP) was measured by UV-VIS spectrophotometer (PerkinElmer, lambda2b, USA), and soil total potassium (TK) was determined by flame photometer (Shanghai Yidian, FP6431, China). DOC was extracted at the soil to distilled water ratio of 1:5 (w/v) in 250r/min shaking for 24h, centrifuged at 4500rpm, filtered through 0.45 μm PES filters, and measured by a TOC analyzer. SBD was measured by the ring knife method, and soil water content (SWC) was measured by drying at 105°C. Soil particle diameter (SPD) was determined using Laser Particle Size Analyzer (Malvern Panalytical, Mastersizer 3000+, Britain). The soil water-stable aggregates were determined by the wet-sieving method described by Cambardella and Elliott (1993) and Huang et al. (2022). Briefly, 50 g of 1 cm sieved soil was transferred into a set of sieves (5 mm, 2 mm, 1 mm, 0.5 mm, 0.25 mm) and submerged in 1 cm of deionized water for 5 min to allow slaking. After slaking, the soil was sieved by moving the nested sieves up and down 40 times/min for 2 min in an aggregate structure analyzer (Laiende, LD-TL100, China), thereby obtaining the particle size distribution of the aggregates. The mean weight diameter (MWD) of soil aggregates was calculated using the following formula:

(Equation 1) $$\text{MWD}=\sum n\;i=1x_{i}w_{i}$$

where X_i_ is the mean average diameter of the different aggregate fractions, W_i_ is the percentage of the different aggregate fractions, n is a different aggregate fraction, i = 1,2,3,4,5,6, representing >5 mm, 2–5 mm, 1–2 mm, 0.5–1 mm, 0.25–0.5 mm, and <0.25 mm, respectively.

#### DNA extraction from soil and high-throughput sequencing analysis

Total genomic DNA samples were extracted using the OMEGA Soil DNA Kit (M5635-02) (Omega Bio-Tek, Norcross, GA, USA), following the manufacturer’s instructions, and stored at −20°C before further analysis. The quantity and quality of extracted DNAs were measured using a NanoDrop NC2000 spectrophotometer (Thermo Fisher Scientific, Waltham, MA, USA) and agarose gel electrophoresis, respectively.

PCR amplification of the bacterial 16S rRNA genes V3-V4 region was performed using the forward primer 338F (5′-ACTCCTACGGGAGGCAGCA-3′) and the reverse primer 806R (5′-GGACTACHVGGGTWTCTAAT-3'). Sample-specific 7-bp barcodes were incorporated into the primers for multiplex sequencing. The PCR components contained 5 μL of buffer (5×), 0.25 μL of Fast pfu DNA Polymerase (5U/μL), 2 μL (2.5 mM) of dNTPs, 1 μL (10 μM) of each Forward and Reverse primer, 1 μL of DNA Template, and 14.75 μL of ddH2O. PCR amplicons were purified with Vazyme VAHTSTM DNA Clean Beads (Vazyme, Nanjing, China) and quantified using the Quant-iT PicoGreen dsDNA Assay Kit (Invitrogen, Carlsbad, CA, USA). After the individual quantification step, amplicons were pooled in equal amounts, and pair-end 2 × 250 bp sequencing was performed using the Illumina NovaSeq platform with NovaSeq 6000 SP Reagent Kit (500 cycles) at Shanghai Personal Biotechnology Co., Ltd (Shanghai, China).

#### Selective dissolution extraction of Fe oxides

Total iron (Total Fe) was wet-digested with an acid solution mixture of HNO_3_-HClO_4_-HF (Duan et al., 2020). Fed, Feo, and Fep were extracted using a solution of dithionite-citrate-bicarbonate (DCB), a solution of ammonium oxalate-oxalic acid, and a solution of sodium-pyrophosphate, respectively.^43^ The amount of Total Fe, Fed, Feo, and Fep in the extracts was measured using phenanthroline spectrophotometry (Shimadzu, UVmini-1240, Japan). The Fe-free index, Fe-activation index, and Fe-complexing index were calculated using the following equations:^11^

(Equation 2) Fe-freeindex(%)=Fed/TotalFe×100%

(Equation 3) Fe-activationindex(%)=Feo/Fed×100%

(Equation 4) Fe-complexingindex(%)=Fep/Fed×100%

#### Fe-bound OC measurements

The Fe-OC contents were determined using the dithionite–citrate–bicarbonate (DCB) extraction method.^31^ In brief, 0.5 g of soil sample was mixed with a buffer solution containing 0.11 M sodium bicarbonate and 0.27 M trisodium citrate (pH 7.3) and heated to 80 °C in a water bath, and 0.5 g of sodium dithionite was added. To eliminate the background release of OC during the heating process, a control soil sample was extracted using sodium chloride (NaCl) solution at the same ionic strength as the DCB solution. Subsequently, mixtures were separated by centrifugation at 4000×g for 20 min. Residues were then rinsed five times with 20 mL of deionized water and freeze-dried. The SOC in residues was analyzed using a TOC analyzer. Fe-OC, *f*_Fe-OC_ (%), and the C:Fe molar ratio of Fe-OC were calculated as:

(Equation 5) $$\text{Fe}-\text{OC}\;\left(g\cdot {\text{kg}}^{-1}\right)={\text{OC}}_{\text{NaCl}}-{\text{OC}}_{\text{DCB}}$$

(Equation 6) $$f_{\text{Fe}-\text{OC}}\;\left(\%\right)=\text{Fe}-\text{OC}/\text{SOC}\times 100\%$$

(Equation 7) C:FeMolarratioofFe-OC=(Fe-OC/MC)/(Fed/MFe)

where OC_NaCl_ and OC_DCB_ were the organic carbon contents in the NaCl and DCB-extracted soil residues, respectively; SOC was the soil organic carbon; MC and MFe represent molar masses of C and Fe, respectively.

#### Measurements of carbon isotopes in SOC and Fe-OC

The stable carbon isotopic ratios (δ^13^C) were determined on a Delta Plus XP mass spectrometer (Thermo Scientific, Bremen, Germany) coupled with an elemental analyzer (FlashEA1112; CE Instruments, Wigan, UK) in continuous flow mode. The standard deviation for measurements of δ^13^C was approximately 0.15‰.

Before the analysis of carbon isotopes, all samples were decarbonated using 0.5M hydrochloric acid. The δ^13^C of Fe-OC was obtained from the following isotopic mass balance equation:

(Equation 8) $$\delta^{13}C_{\text{Fe}-\text{OC}}=\left(\delta^{13}C_{\text{NaCl}}\times 100-\delta^{13}C_{\text{DCB}}\times \left(100-f_{\text{Fe}-\text{OC}\%}\right)\right)/f_{\text{Fe}-\text{OC}\%}$$

where δ^13^C_Fe-OC_ is the δ^13^C of Fe-OC; δ^13^C_NaCl_ and δ^13^C_DCB_ are the δ^13^C of soil treated with NaCl and DCB, respectively.

#### ATR-FTIR analysis

The functional groups of OC_NaCl_ and OC_DCB_ were identified by Attenuated total reflection Fourier transform infrared spectrometry (ATR-FTIR). Briefly, soil samples were ground in an agate mortar and subsequently sieved through a 0.053 mm sieve mesh. The samples were then dried at 60°C for 16 h until constant weight and sequentially placed on the FTIR (Bruker, TensorⅡ, USA) for determination. The instrument parameters were set as follows: spectral range 4000–450 cm^−1^, scanning interval 1 mm, resolution 4 cm^−1^, and 64 scans.

#### Statistics analysis

Before the analysis of data, the outliers in the dataset were removed using SPSS 20.0 (SPSS, Chicago, IL, USA). A one-way analysis of variance (ANOVA) was conducted to first assess the effects of amendment measures on various soil variables.That is, the F-ratio test was employed to determine whether there were significant differences in the overall means of the data from different treatment groups. If the F-ratio test is significant, then the Least Significant Difference (LSD) was used for post-hoc pairwise comparisons to further determine whether there are significant differences among the groups. Bivariate Pearson correlation analyses were employed to assess the impact of each biotic and abiotic variable on various iron indices, Fe-OC, and the C: Fe molar ratio of Fe-OC. We performed two-tailed unpaired t-tests to determine whether the impact is significant. A *p* value <0.05 was considered statistically significant. Redundancy analysis (RDA) was conducted to quantify the contribution of each biotic and abiotic variable to these parameters. Analysis of the alpha diversity index is utilized to quantify the richness of microbial species. ANOVA and bivariate correlation analyses were performed using SPSS software (IBM SPSS Statistical 27, IBM SPSS company, USA). RDA was carried out using Canoco 5 (Microcomputer Power, Ithaca, NY, USA). The alpha diversity index analysis was performed utilizing QIIME2 2022.11. In the CK experiment, each indicator was represented by 5 data points, whereas in the T1 and T2 experiment, each indicator was based on 10 data points. Figures were drawn using OriginLab 2022 software.
