# Supplementary material for: Amendment of saline-alkali soils promotes the formation and stability of iron-bound organic carbon
Source: iScience. 2025 Dec 2;29(1):114314. doi: 10.1016/j.isci.2025.114314 (PMC12800629; doi:10.1016/j.isci.2025.114314)
Supplement: Document S1. Figures S1 and S2 [file mmc1.pdf]

iScience, Volume *29*

Supplemental information

Amendment of saline-alkali soils promotes the formation and stability of iron-bound organic carbon

Shuhan Wang, Xueqin Ren, Tairan Zhou, Yun Zhang, Shuwen Hu, and Biao Zhu

Supplemental-information

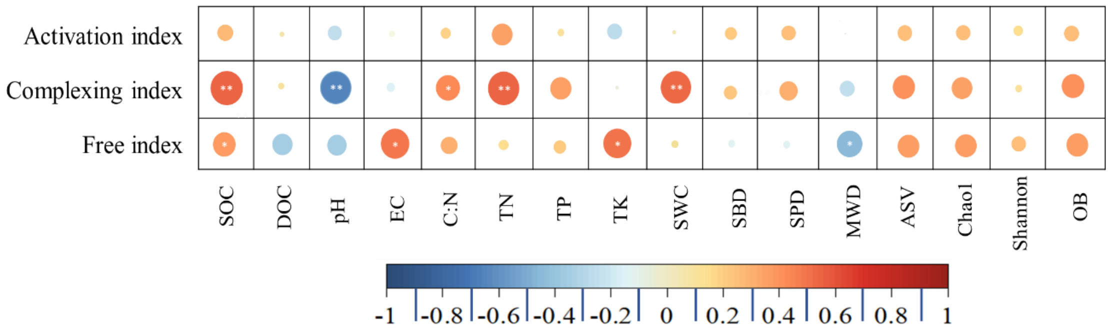

Fig. S1. Pearson correlation analysis of three kinds of iron indices in soil with biotic and abiotic factors. The colors and diameter of each circle are proportional to the value of Pearson's correlation coefficient. The asterisk indicates significance level: * indicate $p < 0.05$ and ** indicate $p < 0.01$

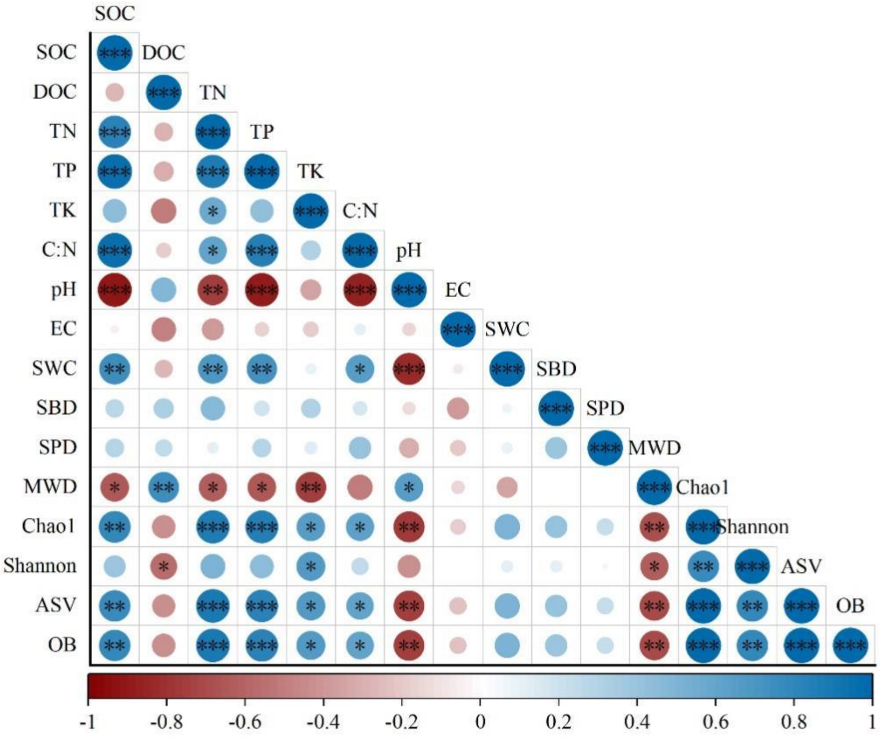

* p<=0.05 ** p<=0.01 *** p<=0.001

Fig. S2. Heat map of Pearson correlation between soil biotic and abiotic variables. The correlation coefficient (r) is represented by a color gradient. * indicates $p < 0.05$ and ** indicate $p < 0.01$
